# Hybrid Membrane Distillation and Wet Scrubber for Simultaneous Recovery of Heat and Water from Flue Gas

**DOI:** 10.3390/e22020178

**Published:** 2020-02-04

**Authors:** Mohd Hizami Mohd Yusoff, Ein K. Nyunt, Muhammad Roil Bilad, Nasrul Arahman, Sri Mulyati, Samsul Rizal, Nik Abdul Hadi Nordin, Jia Jia Leam, Asim Laeeq Khan, Juhana Jaafar

**Affiliations:** 1 Chemical Engineering Department, Universiti Teknologi PETRONAS, Bandar Seri Iskandar, Perak 32610, Malaysia; hizami.yusoff@utp.edu.my (M.H.M.Y.); ingyinnyut@gmail.com (E.K.N.); mroil.bilad@utp.edu.my (M.R.B.); nahadi.sapiaa@utp.edu.my (N.A.H.N.); jiajia.leam@gmail.com (J.J.L.); 2 Department of Chemical Engineering, Universitas Syiah Kuala, Banda Aceh 23111, Indonesia; sri.mulyati@unsyiah.ac.id; 3 Graduate School of Environmental Management, Universitas Syiah Kuala, Jl. Tgk Chik Pante Kulu No. 5, Darussalam, Banda Aceh 23111, Indonesia; 4 Research Center for Environmental and Natural Resources, Universitas Syiah Kuala, Jl. Hamzah Fansuri No. 4, Darussalam, Banda Aceh 23111, Indonesia; 5 Department of Mechanical Engineering, Universitas Syiah Kuala, Banda Aceh 23111, Indonesia; samsul_r@yahoo.com; 6 Department of Chemical Engineering, COMSATS Institute of Information Technology, Lahore 54000, Pakistan; alaeeqkhan@ciitlahore.edu.pk; 7 Advanced Membrane Technology Research Centre (AMTEC), Universiti Teknologi Malaysia (UTM), Skudai 81310, Malaysia; juhana@petroleum.utm.my

**Keywords:** membrane distillation, waste heat recovery, hybrid system, water recovery, flue gas, process intensification

## Abstract

Flue gas contains high amount of low-grade heat and water vapor that are attractive for recovery. This study assesses performance of a hybrid of water scrubber and membrane distillation (MD) to recover both heat and water from a simulated flue gas. The former help to condense the water vapor to form a hot liquid flow which later used as the feed for the MD unit. The system simultaneously recovers water and heat through the MD permeate. Results show that the system performance is dictated by the MD performance since most heat and water can be recovered by the scrubber unit. The scrubber achieved nearly complete water and heat recovery because the flue gas flows were supersaturated with steam condensed in the water scrubber unit. The recovered water and heat in the scrubber contains in the hot liquid used as the feed for the MD unit. The MD performance is affected by both the temperature and the flow rate of the flue gas. The MD fluxes increases at higher flue gas temperatures and higher flow rates because of higher enthalpy of the flue gas inputs. The maximum obtained water and heat fluxes of 12 kg m^−^^2^ h^−^^1^ and 2505 kJm^−^^2^ h^−^^1^ respectively, obtained at flue gas temperature of 99 °C and at flow rate of 5.56 L min^−1^. The MD flux was also found stable over the testing period at this optimum condition. Further study on assessing a more realistic flue gas composition is required to capture complexity of the process, particularly to address the impacts of particulates and acid gases.

## 1. Introduction

Alternative energy sources are required to fulfil the growing demands of energy utilizations. Despite the dominance of the fosil based fuels (crude oil, natural gas and coal) to serve those demands, they are depleting and their utilizations cause detrimental impacts to the environmental [1]. This is one of the major reasons why some countries attempt to explore renewable energy sources that are more benigh to the environment [2] through utilization of the solar energy [3] or the biomass-based fuel to power the transportation sector. Tropical countries like Malaysia and Indonesia have been using biodiesel from palm oil, *Jatropha curcas*, *Ceiba pentandra*, *Reutealis trisperma*, etc. for transportation sector [4,5,6,7,8,9]. One of the other viable options is by energy recovery from the flue gas that contains high energy at relatively low temperature. For energy recovery in the flue gas, high entropy generation due to low temperatures remain a challenge [10].

Flue gas is as a potential thermal energy source to be recovered [11]. Despite considered as the waste heat, it still contains high amount of heat and rich in water moisture [12]. The energy saving potential for this application is very promising. It is estimated that 35 million tons of standard coal globally can be saved annually if half of the latent heat in the flue gas can be recovered [13]. However, the flue gas temperature is relatively low making heat recovery a great challenge [14] because of the large surface area required for energy harvesting. For instance, flue gas from coal-fired power plant generally has temperature below 130 °C, and it contains 10–16% (*v*/*v*) water vapour [15]. To handle such condition, combined cycle for heat recovery or implementation of heat cascade may be necessary [16]. If recovered, the exhaust heat energy from flue gas can be used to reduce heat utilities in plants in expense of the additional capital costs for the energy harvesting infrastructure [17].

In addition to energy, water in flue gas is also worthwhile to be recovered and can be used as the process water. Majority of water supplied to a power plant is used as the boiler feed, as the cooling agent and for the cleaning purposes [18]. Direct emission of exhaust flue gas into the air—with insufficient recovery technologies—would waste both heat and water rendering a power plant to be inefficient. A considerable amount of energy could be recovered when efficient recovery system is implemented.

Technologies for heat recovery from moist gas are well established, with the most popular ones being the organic Rankine cycle (ORC), the absorption systems, the waste heat boilers and the heat exchangers [19]. Comparing these technologies, ORC requires high energy consumption, while absorption involves high regeneration costs, making these two less attractive [20]. In the heat exchangers, latent heat is recovered through the exchange of heat between the moist gas and the cooling medium [21] and judging from the relatively low temperature, it requires a large heat transfer area. Interests are now shifted towards techniques that recover both mass and heat as they offer better heat recovery yield [22].

Several methods have been proposed to recover heat and water from flue gas [23], among them are membrane based processes. Membrane condenser (MC) and transport membrane condenser (TMC) have been proposed for simultaneous water and heat recovery due to their resistance against corrosion and fouling [24], in addition to other common economizer. MC exploits the hydrophobic nature of the membrane to recover water from the flue gas. In MC, flue gas flow is sent into a hydrophobic membrane allows water vapour to pass through and condensed in the permeate side [25]. In MC, up to 60% of water moisture could be recovered. However, despite offering rather high water-recovery, MC process is rather complex. In a full set-up, it involves heat exchanger for lowering the flue gas temperature before it is sent to an MC for condensation.

In TMC, a tubular ceramic membrane is used to recover the condensed water from a flue gas [26,27]. In this system, saturated flue gas is flown to the lumen side of the membrane, while the coolant water is flown in the shell side. The water vapour transport and condensed to the shell side due to vapour pressure differenece. Liquid water is then recovered as the permeate and the energy is recovered as latent heat of the condensing vapor in the shell side [28]. The mechanism of water and heat recovery is similar to the one in membrane distillation (MD) [29,30,31]. Apart from the MC and the TMC, to our best knowledge, no other system involving membrane process for simultaneous water and heat recovery found in the literature.

In this study, we propose and assess performance of a new hybrid process that combines water scrubber and MD for simultaneous recovery of water and heat from flue gases. Unlike the MC and the TMC, the proposed system decouples of water and heat recovery indirectly via a water scrubber unit. Firstly, we evaluate the developed system by assessing its desirable operations and constrains. Later, the effects of flue gas temperature and flow rate on the heat and water recovery by system are also investigated.

## 2. Materials and Methods

### 2.1. Experimental Set Up of Hybrid MD-Wet Scrubber

**Figure 1:**
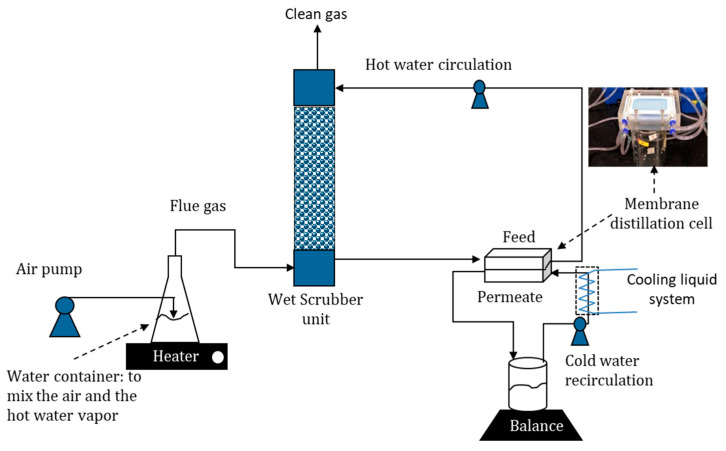
Schematic diagram of hybrid unit that combines wet scrubber and MD cells. The wet scrubber condenses the water vapor from the flue gas and the MD recovers water and latent heat to the recirculated cold water.

A hybrid system comprising of the direct contact MD (DCMD) combined with a wet scrubber column was developed. The hybridization is illustrated in Figure 1. In the sytem, the scrubber firstly recovers the latent heat and the water vapour from the flue gas by contacting the liquid water sprayed from the top to the flue gas entered from the bottom. The water recovery was then achieved by condensing the water vapour from the flue gas and the heat recovery originates from the latent heat of the condensing vapor.

For the lab-scale set-up, a conical flask filled with 1.1 L of water was heated on a hot plate at variable temperatures. The air was then pumped into the hot water at variable rates to produce mixture of air and water vapour treated as the artificial flue gas. The artificial flue gas It resembles the typical temperature of the flue gas from coal-fired power plant and the target industrial incinerator unit [32]. However, the water compositions were twice higher than the typical composition and it did not contain acid gasses and particulate matter due to practical limitations of the setup. A thermocouple was inserted into the flask to measure the temperature of the flue gas coming out from the flask and fed to the wet scrubber unit. Meanwhile, the circulated liquid water flow was sprayed from the top of scrubber to strip the water vapour from the hot flue gas. The dehumidified flue gas flow exited from the top while the hot water flow was circulated as the feed of the MD unit. Cool water flow of 25 °C was passed through the permeate side of the DCMD module at a constant flow rate of 8.34 L h^−1^. As the two flows with different temperatures passed through the MD cell, separated by the polytetrafluoroethylene (PTFE) membrane, vaporization of water vapour and vapour transport occurred across the membrane. The mass of the circulated cold water was monitored overtime and the change of mass was used as one of the parameters to calculate the mass flux in MD as detailed later.

Each test was run for 100 min with data collections at 10 min interval. The recorded parameters were flue gas temperature, stripped flue gas temperature, the temperature of circulating water (hot water flow) and the cold-water mass. Those tests were repeated for different heating values (heater temperatures) and different flue gas flow rates. The heating rate was fixed at the hot plate temperature of 130, 140, 150 and 160 °C at a constant flow rate of 5.56 L min^−1^. Another set of conditions with flue gas flow rates of 2.62, 5.56 and 7.61 L min^−1^ at constant plate temperature of 150 °C were also run. The flue gas flowrates and temperatures were selected based by considering the set-up limitation. The construction material could not withstand temperature >90 °C and too high of flow rate lead to excessive leakage. Nonetheless, the parameters met the basic process parameters requirement. The temperature of the MD feed must be >40 °C to allow high MD flux. The temperature of the hot liquid water must also be lower than 90 °C to be compatible with the scrubber material. The scubber unit was constructed from poly(methyl methacrylate) material and during the trial-and-error of the set-up prior the experiment, it was noticed that it could not withstand temperatures above 90 °C which would lead to leakages on the joints and the connections.

Two parameters were chosen to access the overall system performance in this study. Firstly, the flue gas temperature was varied from 84 °C to 99 °C by varying the heater temperature from 130 °C to 160 °C. In this test, the flue gas flow rate was kept constant at 5.56 L min^−1^. Secondly, the flue gas flow rate was varied from 2.63 to 7.61 L min^−1^ at a constant heater temperature of 150 °C. The range of the flue gas flow rates were chosen based on practical consideration. At flue gas flow rates of >2.63 L min^−1^, the temperature of the hot liquid water dropped drastically, leading to low MD fluxes. For flue gas flow rates of >7.61 L min^−1^, the hot water container could not withstand the high pressure leading to leakages.

### 2.2. Membrane Characteristics

The membranes used in this experiment was the commercial porous hydrophobic PTFE membrane from milipore with the properties summarized in Table 1. The PTFE membranes is known to be applicable for MD in term of porosity, tortuosity and pore size, with good performance [33]. It has a low surface energy of 9.1 × 10^−3^ N m^−1^.

### 2.3. Scrubber Unit Installation

A wet scrubber set up had 6 cm in diameter and 29 cm in height. The scrubber was a packed column, filled with gravels of 0.5 cm diameter that facilitate contacts between the flue gas, and the circulated water: the hot flue gas entered from the bottom and circulated liquid water flow entered the column and sprayed through the top. The resulted bottom hot water flow exiting from the wet scrubber was a mixture of the circulated water and the condensed water from the flue gas.

### 2.4. Membrane Distillation Cell Assembly

Membrane filtration cell was equipped with a rectangular holder mounted with a membrane sheet that had an affective area of 34 cm^2^ (8.5 × 4 cm). A spacer was in each side of the membrane sheets to allow hot and cold-water flow. The MD process was operated under DCMD configuration. The hot liquid flow from the bottom of the wet scrubber unit was used as the feed. In the MD cell, vaporization of water and vapour transport across the membrane occurred. A mass balance was used to measure the water recovery rate on permeate side as detailed elsewhere [34].

### 2.5. Fluxes and Recovery Determination

Recovery of water in the scrubber column was measured experimentally by condensing the moisture content of the feed flue gas and the clean gas, the flue gas exiting from the wet scrubber. The moisture content of the feed and the output flue gases were fully condensed using a cold trap. The percentage of water recovery (%R) was then calculated using Equation (1).

(1) $$\%R=\frac{Cd_{v}-Cd_{s}}{Cd_{v}}\times 100\%$$

where $Cd_{v}$ is water condensation rate (kg h^−1^) from the hot water container and $Cd_{s}$ water condensation rate (kg/h) of the flue gas coming out from scrubber column.

The energy recovery from the flue gas in the wet scrubber was calculated from the specific enthalpy data obtained from the steam table, as depicted in Equation (2). The method of estimatinng the energy recovery from the flue gas neglected the heat-loss due to conduction. It then follows that all the recovered heat to be contained by the hot water that acted as the feed for the MD system. The energy in the hot liquid was then further transferred into the cold liquid for further usage (i.e., pre-heated of a boiler unit).

(2) $$\%q_{R}=\frac{m_{FG}h_{FG}-m_{CG}h_{CG}}{m_{FG}h_{FG}}\times 100\%$$

where $\%q_{R}$ is the percentage of heat recovery (%), $m_{FG}$ the mass of the flue gas (kg h^−1^), $h_{FG}$ the specific entalphy of the flue gas (kJ kg^−1^), $m_{FG}$ the mass of the flue gas (kg h^−1^), $h_{FG}$ the specific entalphy of the clean gas (kJ kg^−1^).

Two main performance parameters obtained from the MD unit were the mass and the heat fluxes. The mass flux obtained was calculated according to Equation (3).

(3) $$J_{m}=\frac{\Delta \omega }{A.\Delta t}$$

where Δω is the change in permeate water weight (kg) during a time period ∆*t* (h), *A* (m^2^) and *J_m_* (kg m^−2^ h^−1^) are the effective membrane area and mass flux, respectively. The heat flux was calculated based on mass flux as in Equation (4).

(4) $$q_{m}=J_{m}\Delta h_{v}$$

where *q_m_* is heat flux obtained (kJ m^−2^ h^−1^) and ∆*h_v_* the specific enthalpy different of water inlet and outlet (kJ kg^−1^) at corresponding temperatures. It is worth noting that the conductive heat transfer was neglected in this study since the value is much smaller than the convective heat transfer through the condensation of water vapor permeate. It is also based on the assumption that the full-scale unit is very well insulated.

## 3. Results and Discussion

### 3.1. Hybrid System Operation Assessment

**Figure 2:**
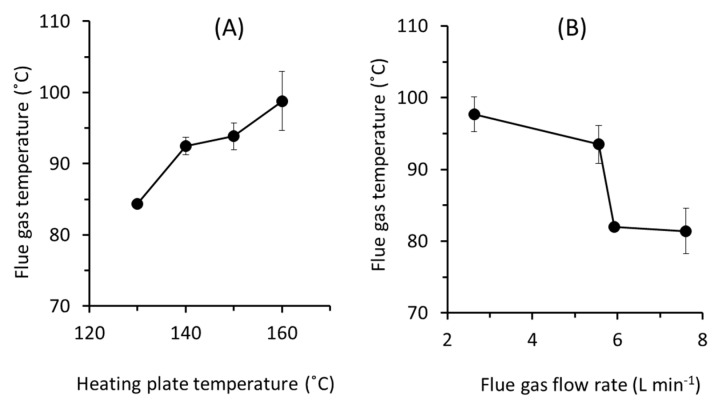
Effect of (**A**) heater temperature at a fixed flue gas flow rate of 5.56 L min^−1^ on the flue gas temperature and (**B**) the effect of flue gas flow rate at a constant heating rate of 150 °C.

The tread-off between the flue gas flow rate and heating temperature is shown in Figure 2. It illustrate the span of operational parameters applicable for the developed system. The water vapour contents in the flue gases were about constant at 57.0, 57.1, 58.7 and 58.9 v% for the flue gas temperatures of 84, 92, 94 and 99 °C. On the other hand, increasing the flue gas flow rate reduces the flue gas temperature and the water contents of 53.4, 39.0, 37.1 and 33.0 v% for flue gas flow rate of 2.63, 5.56, 5.92 and 7.61 L min^−1^, respectively. The decrease in flue gas temperature at high flue gas flow rates is attributed by the lower enthalpy of the flue gas flow due to lesser water vapour fraction generated a constant heating rate. Maintaining the heating plate temperature lead to constant heat supply to the system, and consequently the total volume of the water vapour did not change significantly. When increasing the air supply flowrate, the composition of the water vapour decreased. As detailed earlier, the composition of water vapor drops from 54.3 v% to 33.0 v% when the total flue gas flow rate increases from 2.63 to 7.61 L min^−1^, respectively. The corresponding volumetric flow rate of water vapour are 1.43 and 2.51 L min^−1^ for the flue gas flow rate of 2.63 and 7.61 L min^−1^, respectively.

### 3.2. Performance of the Scrubber Unit

**Figure 3:**
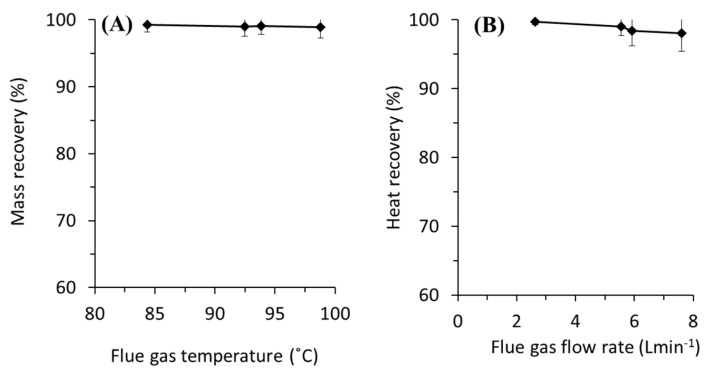
(**A**) Recovery of water as a function of the flue gas temperature and the flue gas flow rate run at the flue gas flow rate of 5.56 L min^−1^. (**B**) Recovery of heat as a function of the flue gas flow rate run at the flue gas temperature of 94 °C.

The scrubber unit achieves almost complete water and heat recovery (Figure 3) irrespective of the flue gas temperatures and the flow rates. Increasing the flue gas flow rate and temperature slightly decreases the water and heat recovery. It is worth noting that the heat recovery calculation here assumes no conductive heat-loss and the actual value should be less that the one reported. More accurate recoveries can be obtained by calculating the mass-energy balance of the system, which was found difficult because of the dynamic condition of the system. These findings demonstrate the effectiveness of the water scrubber in stripping the water moisture and capturing the latent heat. It turns the water phase from hot vapour to hot liquid, which later used as the MD feed.

It is worth noting that liquid water temperature in this system can be controlled by balancing of MD permeate and make-up cold water entering the scrubber unit. This way, solubilisation of acid gas present in real flue gas can be avoided by applying higher liquid water temperature beyond the dew point of the acid gases. High temperatures ensure that both heat and water recovery in the scrubber are satisfactory.

### 3.3. Performance of MD Unit

#### 3.3.1. Effect of Temperature

**Figure 4:**
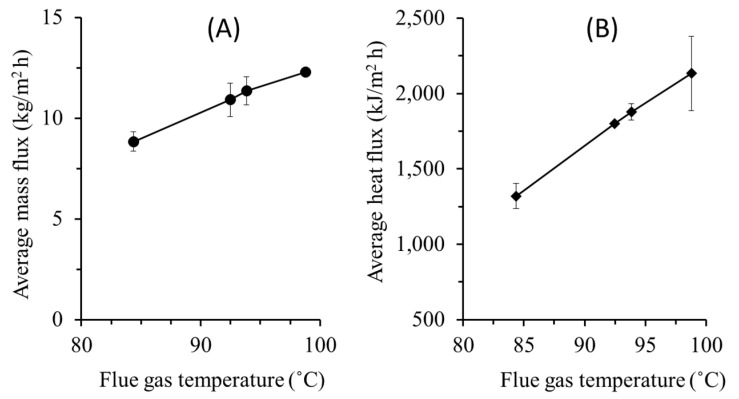
Effect of flue gas temperature on (**A**) water flux, (**B**) heat flux. The tests were run at a constant flue gas flow rate of 5.56 L min^−1^.

Both mass and heat fluxes in MD increase at higher flue gas temperatures (Figure 4). Higher flue gas temperature leads to higher liquid water in the MD feed, and as the cold-water temperature remains the same, it leads to higher driving force that promotes higher mass and heat fluxes. Figure 4A shows the average mass flux increases by 39% as the flue gas temperature rises from 84 to 99 °C. Convective heat transfer across MD occurs in the form of mass transport of the water vapour across the membrane. In MD, the conductive heat transfers through PTFE membrane is considered very small due to small membrane area coupled with high membrane porosity (70%) and low conductivity of PTFE (0.25 W/(m·K)). The fluxes can also be enhanced by applying membranes with better intrinsic properties [35,36,37].

Heat flux also increases by 40% when the flue gas temperature is elevated by 12 °C as shown in Figure 4B. These findings indicate that temperature polarization coefficient drops at higher flue gas temperature. The highest water and heat flux of 12 kg m^−2^ h^−1^ and 2505 kJ m^−2^ h^−1^ were obtained respectively at flue gas temperature of 99 °C and flie gas flow rate of 5.56 L min^−1^.

#### 3.3.2. Effect of Flue Gas Flow Rate

**Figure 5:**
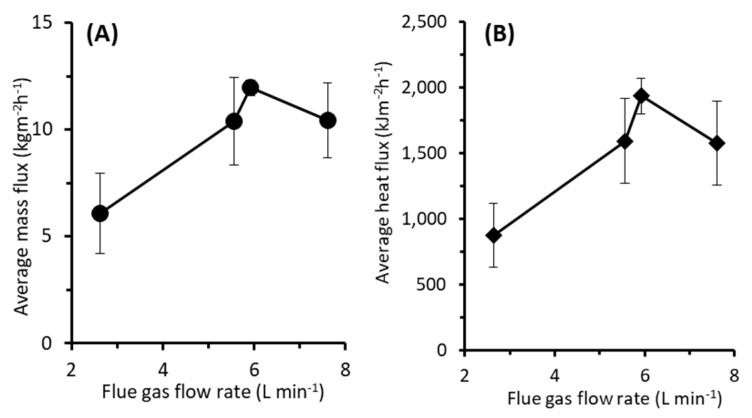
Effect of flue gas flow rate on (**A**) water and (**B**) heat flux. The tests were run at flue gas temperature of 94 °C.

Low flue gas flow rate leads to low water and heat fluxes (Figure 5). The result shows that for the lowest flue gas flow rate of 2.63 L min^−1^, the fluxes are the lowest of 6.07 kg m^−2^ h^−1^ and 875 kJ m^−2^ h^−1^ for mass and heat fluxes, respectively. At a low flue gas rate, excessive heat loss along the tubing line of the MD system occurs. Therefore, the heat recovery in the MD becomes low resulting in low water and the heat fluxes. Increasing the flue gas flow rate to 5.56 and 5.92 L min^−1^ increases the fluxes. However, once the flow rate increases to 7.61 L min^−1^, both mass and heat fluxes drop. This agrees with findings discussed in Section 3.1. Since the flue gas temperature drops at higher flue gas flow rates, the total heat recovered in the scrubber also decreases. This leads to lower liquid feed temperature of MD and eventually decreases the fluxes, as also suggested elsewhere [38].

Increasing the flue gas rate significantly increases the water and heat fluxes (Figure 5). At the maximum flue gas flow rate of 5.92 L min^−1^, the achieved mass and the heat fluxes are 11.95 kg m^−2^ h^−1^ and 1936 kJ m^−2^ h^−1^, respectively. At higher flue gas flow rates, the higher the energy input to the system, and like for the effect of the temperature, it increases the MD fluxes. The mass flux is comparable with the performance TMC and MC systems [30].

#### 3.3.3. Water and Heat Flux Evolution

**Figure 6:**
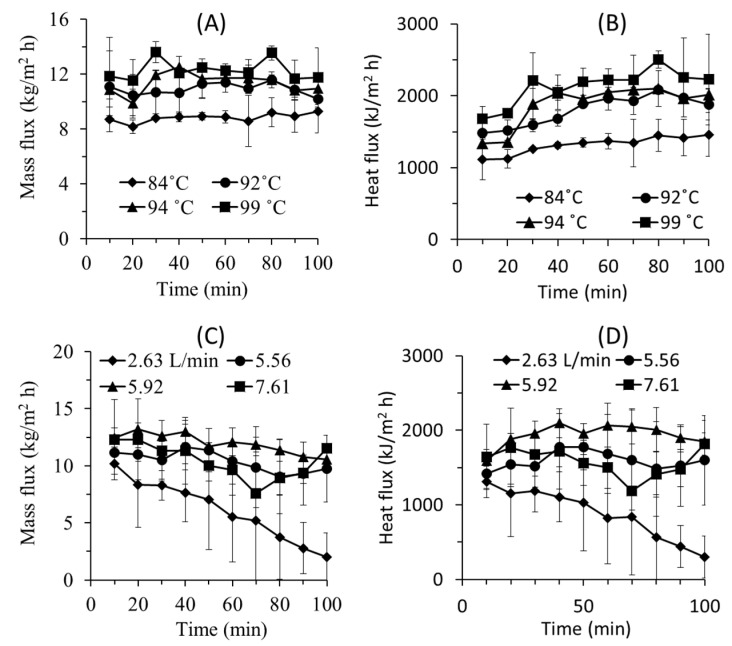
Evolution of flux (**A**) mass and (**B**) heat flux as a function of flue gas temperature, and (**C**) mass and (**D**) heat flux as function of flue gas flow rate.

The system operated under semi-batch mode and still within the unsteady-sate condition. As the operation progress, the cold-water temperature increases to contain the recovered heat leading to water and heat flux decrease overtime for system at low flue gas flow rate (Figure 6). The decrease in the MD feed water temperature lowers the fluxes. Consequently, there is an accumulation of water liquid in the wet scrubber unit. This issue can be avoided in the full-scale application by adjusting the makeup water flow rate and the temperature of liquid in the scrubber unit. The feed MD feed temperature can be controlled by makeup water to obtain constant fluxes.

The effect of flue gas temperature on heat and water fluxes are rather stable over time (Figure 6A,B). The decreasing flux trend over time is noticeable in the lowest applied flow rate of 2.63 L min^−1^. It can be explained by the starting temperature difference between feed and permeate side that is comparably smaller than at the higher flow rates. At the lowest flow rate, the water flux reduces from 10 kg m^−2^ h^−1^ to 2 kg m^−2^ h^−1^.

Figure 6A,B also show that higher flue gas temperature leads to almost stable or an increase in both mass and heat fluxes. This can be explained by the low applied membrane area and water vapour content in the simulated flue gas. The rate of water and heat recovery by MD is lower than the rate of water and energy supply from the flue gas. Therefore, the water and heats accumulate in the scrubber system increasing the water volume and temperature. As consequence higher MD flux as function of time is obtained. At extended operation, ones can imagine that the system will reach a steady state where the water and heat flux in the MD balance the water and heat input to the system from the flue gas.

The fluxes decline as a function of time when the system was run under variable flue gas flow rates (Figure 6C,D). Under the testing conditions, the contributions of membrane fouling and membrane wetting in declining the flux are are low because of the use of pure water and short experimental duration [37,39]. However, flux decline over time is obvious when flue gas flow rate was varied. When comparing Figure 6A,B with Figure 6C,D, the results suggest that the flue gas flow rate is more dominant in affecting the MD performance than the temperature. The declining trend of the flux can be explained by the unsteady state of the system operating under semi-batch. At low flowrate, the supply of the flue gas is too low to maintain the hot water temperature overtime. Therefore, the temperature of the hot water decreases overtime leading to lower mass and heat fluxes.

### 3.4. Practical Consideration

Experimental results show that MD performances decrease with the rise in gas flow rates. At higher flue gas rate, the water content is less resulting in lower enthalpy content. However, real flue gases in a steady state operation have almost constant enthalpy in the flow. Thus, higher flue gas flow rates leads to higher total energy content which increases the hot water temperature and eventually increase the water and heat flux of the MD.

The developed system can be of use for separating particulate matter present in the flue gas flow in addition to recovering the heat and the water. Hot flue gas normally consists of impurities such as NO*_x_* and SO*_x_* which can be soluble under their dew point temperature. Thus, it is important to keep the flue gas temperature above the lowest dew point of those acids. The scrubber not only can be used to recover the mass and the heat from flue gas, but also can entrap particulate matters present in the real flue gas and can be used to control water temperature.

Membrane area is also a critical attribute to the MD toward the overall system performance. It can be the limiting factor to high MD heat fluxes. Larger effective membrane area could be used to achieve higher water vapor transport. However, this lead to lower permeate flux in long run due to the decrease in the temperature difference across the membrane. In the proposed system, the issue could be overcome by adjusting the temperature of the make-up water to the scrubber unit to obtain more stable and higher MD fluxes.

## 4. Conclusions

This study develops a new hybrid process comprising of a wet scrubber and MD for simultaneous recovery of low-grade heat and water from flue gas. The overall performance is dictated by the MD unit since the scrubbing unit recovers most of water and heat. The MD performance is affected by the flue gas temperature and the flow rate. The MD fluxes increases at higher flue gas temperature because of higher enthalpy of the input contained by the hot water for MD feed. The same reason explain the increasing flux at higher flue gas rate. The overall system was more affected by changes in flue gas flow rate than the temperature. The system obtained highest MD flux of 12 kg m^−2^ h^−1^ and heat flux of 2505 kJ m^−2^ h^−1^ at the flue gas flow rate of 5.56 L min^−1^ and the flue gas temperature of 99 °C. This study only unravel few basic parameters of the hybrid wet scrubber and MD unit. Many aspect in the real flue gas were ignored, such as the presence of the acid gasses and particulate matter that may be sensitive for the system operation. Those aspects must be addressed in the future study. Moreover, upon availability of the data cost-benefit analysis need to be performed to gauge the economic potential of the proposed system.

## Figures and Tables

**Table 1:** Properties of membrane used as provided by the supplier.

Physicochemical Information	Value
Pore Size (µm)	0.22
Air flow rate (L min^−1^ cm^2^)	3
Bubble Point (psi)	14.8–20.9
Porosity (%)	70
Filter Surface	Plain
Thickness (µm)	175
Cutting dimension (cm^2^)	8.5 × 4
